# Viral etiology of severe acute respiratory infections in hospitalized patients, Shandong, China

**DOI:** 10.1371/journal.pone.0328439

**Published:** 2025-07-29

**Authors:** Chunwang Li, Yujie He, Xiao Pan, Huimin Yin, Yaowen Pei, Shaoxia Song, Lin Sun, Shu Zhang, Julong Wu, Zhong Li, Xianjun Wang, Zengqiang Kou, Lei Zhao, Weijia Xing, Ti Liu

**Affiliations:** 1 School of Public Health, Shandong First Medical University, Jinan, Shandong, China; 2 Shandong Center for Disease Control and Prevention, Shandong Institute of Preventive Medicine, Shandong Provincial Key Laboratory of Intelligent Monitoring, Early Warning, and Prevention of Infectious Diseases (in preparation), Jinan, China; 3 Shandong Institute of Parasitic Disease Control, Shandong First Medical University (Shandong Academy of Medical Sciences), Jining, Shandong, China; 4 School of Public Health, Shandong Second Medical University, Weifang, Shandong, China; 5 Shouguang Center for Disease Control and Prevention, Weifang, Shandong, China; Cairo University Faculty of Veterinary Medicine, EGYPT

## Abstract

**Background:**

Severe Acute Respiratory Infection (SARI) represents a critical global public health challenge, accounting for substantial severe morbidity and hospitalization burdens with distinct geographical patterns in etiological profiles. This study systematically characterizes the epidemiological and clinical phenotypes of SARI patients in Shouguang county, Shandong Province, China.

**Methods:**

A prospective observational study was conducted at Shouguang People’s Hospital between August 28, 2023 and April 30, 2024, enrolling 1,730 hospitalized patients with SARI from the Departments of Infectious Diseases and Respiratory and Critical Care Medicine. Standardized electronic case report forms were used to systematically collect the demographic characteristics, clinical manifestations and laboratory testing results. Oropharyngeal swab specimens were collected within 24 hours of admission for each patient and stored at −80°C. Multiplex real-time quantitative PCR (RT-qPCR) was performed using the ABI 7500 system to detect 11 respiratory viruses infection, including influenza A virus (IFA), influenza B virus (IFB), respiratory syncytial virus (RSV), parainfluenza virus (HPIV), human coronaviruses (HCoV), human metapneumovirus (HMPV), rhinovirus (HRV), enterovirus (EV), human bocavirus (HBoV), human adenovirus (HAdV), and SARS-CoV-2 (COVID-19).

**Results:**

501 samples (28.96%) were tested positive for at least one virus. The most frequently detected viruses and their infection rates were as follows: IFA (11.33%), COVID-19 (6.53%), HPIV (2.31%), HCoV (2.20%), RSV (1.79%), IFB (1.68%), HMPV (1.56%), EV (0.64%), HADV (0.52%), and HBoV (0.06%). Among patients aged 0–14 years, IFA and EV had the highest infection rates, both at 9.46% (7/74). In the 15–24 age group, IFA exhibited the highest infection rate at 19.70% (26/132). In patients aged ≥70 years, COVID-19 was the most frequently detected virus, with a infection rate of 10.69% (65/608). The overall virus infection rate peaked at 60.00% (30/50) in epidemiological week 48 of 2023. During weeks 46–50 of 2023, the overall infection rate remained consistently high (range: 28.42–60.00%). Significant differences in infection rates were observed across hospital departments (χ² = 5.52, *P* < 0.05), The Department of Infectious Diseases demonstrated a higher infection rate of 34.91% (162/464) compared to 29.07% (368/1266) in the Department of Respiratory Medicine.

**Conclusion:**

Viral etiological analysis of SARI patients in Eastern China identified IFA, COVID-19, and HPIV as the three predominant virus, with influenza virus exhibiting the highest frequency of co-infection with other respiratory viruses. Our study further revealed significant heterogeneity in virus distribution across different hospital departments, age groups, and admission periods. The most common clinical manifestations were cough and fever, with distinct symptomatic profiles observed among infections caused by different viruss. These findings provide scientific evidence to inform government strategies for optimizing the prevention and management of respiratory infectious diseases.

## Introduction

Severe acute respiratory infections (SARI) represent a prevalent clinical entity and constitute a major contributor to global morbidity and mortality [1]. The burden is particularly high among children, the elderly, and immunocompromised individuals [2]. Annually, severe acute respiratory epidemics result in 3–5 million severe cases worldwide, with associated deaths ranging from 290,000–650,000 [3]. Lower respiratory infections, together with COVID-19, constitute the most lethal communicable diseases.SARI can be caused by various pathogens, including viruses, bacteria, and fungi. The primary viral agents responsible for SARI include respiratory syncytial virus (RSV), parainfluenza virus (HPIV), influenza A virus (IFA), influenza B virus (IFB), and human adenovirus (HAdV) [4]. Strict diagnostic criteria are essential for determining the causative pathogens in SARI cases; however, empirical antibiotic treatment is frequently administered in clinical settings. The overlapping clinical presentations of SARI make it challenging to reliably distinguish between viral and bacterial infections based on symptoms alone [5].

The infectivity of respiratory viruses varies significantly across different regions, which may be associated with climatic, cultural, and geographical factors [6].Particularly in China – a populous country with diverse climate zones, varying population densities, and distinct urban-rural disparities – these factors may contribute to unique viral transmission patterns.Therefore, continuous surveillance and understanding of the epidemiological characteristics of respiratory viruses in a specific region are crucial for controlling SARI. Comprehensive epidemiological data on respiratory virues in China remain scarce, with most existing studies focusing exclusively on pediatric populations.This critical data gap substantially constrains the development of precision prevention strategies, particularly for adult populations and ecotone regions. Given that the transmission dynamics of respiratory viruses are influenced by multifactorial environmental determinants including climatic conditions and population mobility, systematic surveillance in demographically representative regions is crucial. This study analyzed 1,730 respiratory specimens collected from SARI patients (2023–2024) to elucidate the circulating patterns and environmental drivers of respiratory viruses in East China’s temperate monsoon climate zone. Our findings provide critical scientific evidence for developing precision prevention strategies against SARI.

## Materials and methods

### Study population and design

We conducted a prospective observational study enrolling consecutive hospitalized patients meeting the diagnostic criteria for SARI at the Departments of Respiratory and Critical Care Medicine and Infectious Diseases, Shouguang People’s Hospital, Shandong Province, China, between August 28, 2023 and April 30, 2024.

SARI diagnosis aligned with the definition outlined by the World Health Organisation [7], the presence of severe acute respiratory infection symptoms,along with a history of fever (≥38°C), new onset of cough or worsening of pre-existing cough, necessitating hospitalisation.

A prospective observational study of 1,730 SARI patients was conducted at Shouguang People’s Hospital (August 2023-April 2024). Using standardized e-forms, we collected demographic, clinical and laboratory data. Oropharyngeal swabs collected within 24h of admission (−80°C storage) were tested for 11 respiratory viruses (IFA, IFB, RSV, HPIV, HCoV, HMPV, HRV, EV, HBoV, HAdV, COVID-19) via RT-qPCR (ABI 7500).

Written informed consent was obtained from all adult participants and legal guardians of minor participants.This study strictly adhered to the ethical principles for medical research involving human subjects as outlined in the Declaration of Helsinki. It was approved by the Preventive Medicine Ethics Committee of the Shandong Center for Disease Control and Prevention (Approval No.: (2021−24).

### Sample collection and laboratory processing

For all enrolled SARI cases, oropharyngeal swab specimens were collected within 3 days of symptom onset using sterile viral transport tubes containing viral transport medium. Immediately after collection, specimens were stored at 4°C and transported on the same day to the Shouguang Center for Disease Control and Prevention (CDC). At Shouguang CDC, specimens were preserved at −80°C until weekly batch transportation under cold chain conditions to Shandong Provincial CDC for analysis.

At Shandong Provincial CDC, viral RNA was extracted and analyzed by multiplex real-time quantitative PCR (RT-qPCR) using the Xi’an Tianlong Respiratory Virus Nucleic Acid infection Kit (Catalog No.: YP2010). This multiplex PCR assay simultaneously detected 11 respiratory viruses infection: IFA, IFB, RSV, EV, HPIV, HBoV, HAdV, HCoV, HMPV, HRV, and COVID-19. All testing procedures strictly followed the manufacturer’s protocols.

### Statistical analysis

Statistical analyses were performed using SAS software (version 9.4 or higher; SAS Institute Inc, Cary, NC, USA). Descriptive frequencies were presented as mean ± standard deviation and proportions. Chi-square tests and Fisher’s exact tests were used for comparisons between groups in terms of categorical variables wherever appropriate. Multivariate logistic regres- sion analysis was performed to identify factors associated with the outcomes of interest. Graphs and visualizations were generated using GraphPad Prism 9 (GraphPad Software, San Diego, CA, USA). A *P*-value < 0.05 was considered statistically significant.

## Results

### General characteristics of enrolled patients

1,730 hospitalized cases with SARI were recruited in our study. Cases were predominantly recruited from the respiratory department 1,266 (73.18%) cases and infectious disease department 464 (26.82%) cases. Among them, 985 (56.94%) males and 745 (43.06%) females, with a mean age of 57.2 ± 21.8 years. The age stratification was as follows: 74 cases (4.28%) aged 0–14 years, 132 (7.63%) aged 15–24 years, 565 (32.66%) aged 25–59 years, 351 (20.29%) aged 60–69 years, and 608 (35.14%) aged ≥70 years. The most frequent clinical manifestations included cough 1,503 cases (86.88%), fever 1,026 (59.31%), sore throat 279 (16.13%), dyspnea 152 (8.79%), nasal congestion 66 (3.82%), diarrhea 45 (2.60%), and pulmonary rales 119 (6.88%). (Table 1).

**Table 1:** Baseline demographic characteristics of hospitalized patients with severe acute respiratory infections.

Characteristic	Virus positive n (%)	Virus negative n (%)	Overall n (%)	χ^2^	*P*
Sex				0.603	0.438
Male	278 (28.22%)	707 (71.78%)	985 (56.94%)		
Female	223(29.93%)	522 (70.07%)	745 (43.06%)		
Age group (years)				9.701	0.046^*^
<15	24 (32.43%)	50 (67.57%)	74 (4.28%)		
15 - 25	50 (37.88%)	82 (62.12%)	132 (7.63%)		
25 - 60	169 (29.91%)	396 (70.09%)	565 (32.66%)		
60 - 70	85 (24.22%)	266 (75.78%)	351 (20.29%)		
≥70	173 (28.45%)	435 (71.55%)	608 (35.14%)		
Department				5.515	0.019^*^
Respiratory	347 (27.41%)	919 (72.59%)	1266 (73.18%)		
Infectious Diseases	154 (33.19%)	310 (66.81%)	464 (26.82%)		
All	501 (28.96%)	1229 (71.04%)	1730 (100.00%)		

### Etiology and epidemiology of respiratory virus

**Fig 1:**
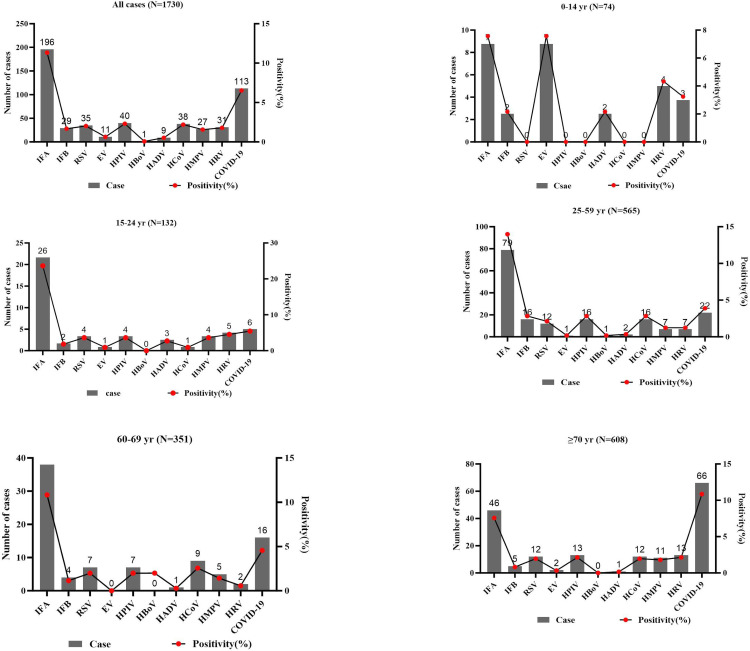
Overall infection Rates and Age-Specific Distribution of Respiratory Virus.

A total of 501 patients (28.96%) tested positive for at least one respiratory virus. Among the virus-positive cases, 69.26% (347/501) were from the respiratory department and 30.74% (154/501) from the infectious disease department, with statistically significant differences between departments (χ² = 5.52, *p* = 0.019). Males accounted for 55.49% (278/501) and females 44.51% (223/501), showing no statistically significant gender difference (χ² = 0.60, *p* = 0.438). Age distribution was 4.79% (24/501) for 0–14 years, 9.98% (50/501) for 15–24 years, 33.73% (169/501) for 25–59 years, 16.97% (85/501) for 60–69 years, and 34.53% (173/501) for ≥70 years, with statistically significant differences among age groups (χ² = 9.70, *p* = 0.046) (Fig 1).

**Fig 2:**
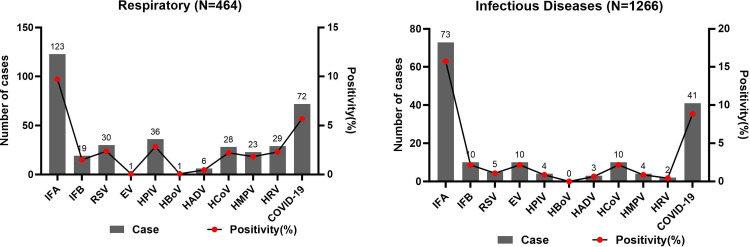
Virus infections between respiratory and infectious disease departments of hospitals).

Among respiratory department patients, IFA was the dominant virus, followed by COVID-19 and HPIV. Among infectious disease department patients, IFA infection was also the most common, followed by COVID-19 and IFB infections (Fig 2).

### Weekly temporal patterns of respiratory virus circulation

**Fig 3:**
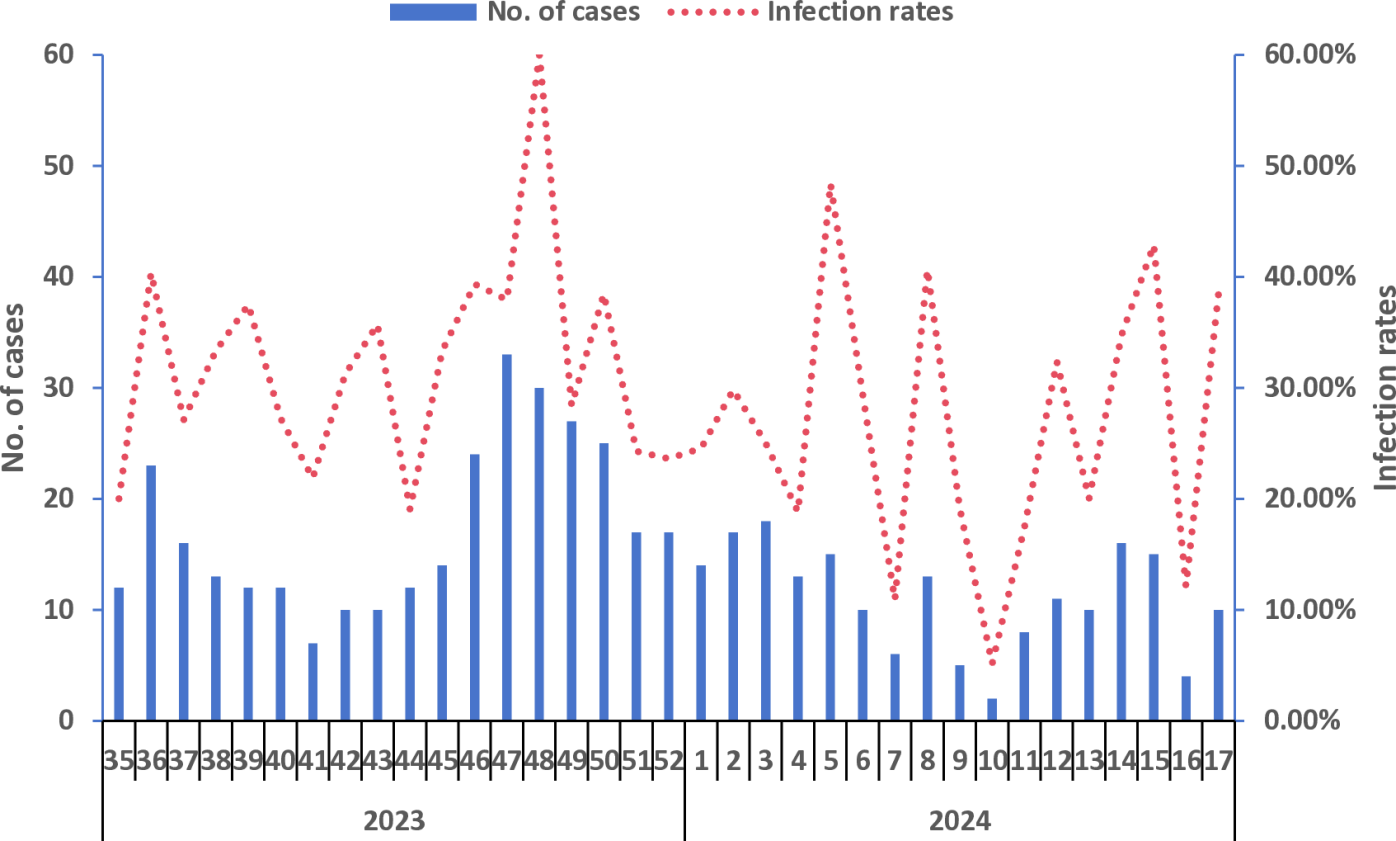
Weekly Trends in Respiratory virus Infected Cases and Infection Rates.

Between the 35th week of 2023 and the 17th week of 2024, multiple respiratory viruses demonstrated year-round co-circulation with distinct seasonal peaks for different viruses. The highest infection rates occurred in week 48 of 2023 (60.00%, 30/50) and week 5^th^ of 2024 (48.39%, 15/31). Notably, the number of detected respiratory virus remained high from the 46^th^ to the 50^th^ week of 2023 (Fig 3).

**Fig 4:**
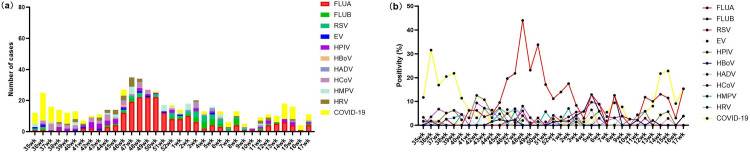
Weekly Distribution of 11 Respiratory Virus. **(a) Number of Virus Detected, (b) Virus Infection Rates**).

The highest infection rate for IFA was observed in the 48^th^ week of 2023 44.00% (22/50), while IFB peaked in the 8^th^ week of 2024 12.50% (4/32). COVID-19 demonstrated its peak infection rate during 36^th^ week, 2023 (31.58%, 18/57), while RSV reached its maximum infection in 48^th^ week, 2023 (6.00%, 3/50). Among other respiratory virus, the highest infection rates were observed for: HPIV in 42^th^ week, 2023 (12.50%, 4/32); HCoV in 48^th^ week, 2023 (8.00%, 4/50); HMPV in 2^nd^ week, 2024 (7.02%, 4/57); and HRV in 45^th^ week, 2023 (7.14%, 3/42) (Fig 4).

### Co-infection patterns of respiratory viruses

**Fig 5:**
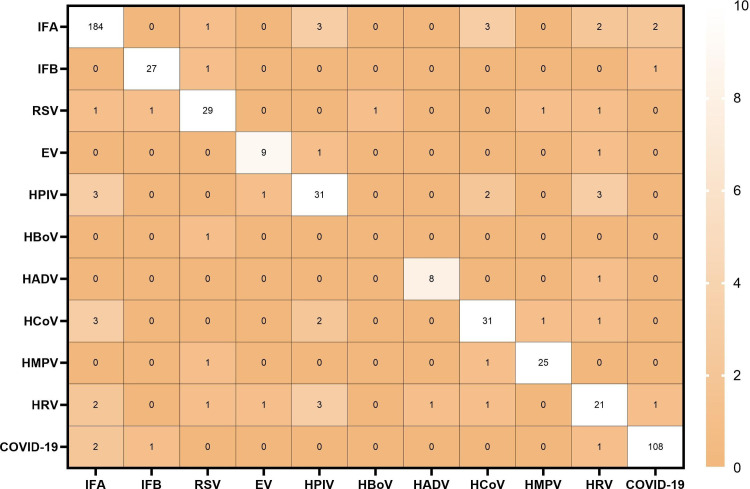
Heat map shows co-infection of two respiratory viruses.

Among 501 SARI patients who tested positive for respiratory viruses, 5.59% (28 cases) had mixed viral infections, including 27 cases of dual infection and 1 case of triple infection. Among the dual infections, IFA co-infection with other viruses was the most common (11/28, 39.29%), followed by HRV co-infection (10/28, 35.71%). The single triple infection case involved IFA, RSV and COVID-19 (Fig 5). Mixed infection cases were distributed across age groups as follows: 0–14 years (1 case, 3.6%), 15–24 years (5 cases, 17.9%), 25–59 years (10 cases, 35.7%), 60–69 years (4 cases, 14.3%), and ≥70 years (8 cases, 28.6%).

### Clinical indicators and symptoms associated with respiratory viruses

Among the 1,730 SARI patients included in the analysis, those with complete clinical records exhibited the following predominant symptoms: cough (88.26%, 1503/1703), fever (59.93%, 1026/1712), sore throat (17.42%, 279/1602), dyspnea (9.55%, 152/1592), pulmonary rales (7.49%, 119/1589), nasal congestion (4.15%, 66/1589), and diarrhea (2.82%, 45/1593).

Symptom-pathogen association analysis revealed that cough was significantly associated with IFA and COVID-19 infections (*P* < 0.05). Specifically, IFA-infected patients showed significantly increased odds of cough (OR = 4.19, 95% CI: 1.97–8.90) compared to non-infected individuals. Both fever and sore throat were significantly associated with IFA infection (*P* < 0.05), while nasal congestion was significantly linked to influenza B virus (IFB) infection (*P* < 0.05). Pulmonary rales showed significant associations with both IFA and COVID-19 infections (*P* < 0.05), with COVID-19 patients had significantly higher odds of severe disease (OR = 4.16, 95% CI: 2.40–7.23). In contrast, respiratory RSV, HPIV, HCoV, HMPV, and HRV infections demonstrated no statistical correlations with these clinical symptoms (*P* > 0.05). (Table 2).

**Table 2:** Multivariate Logistic Regression Analysis of Clinical characteristics associated with respiratory viruses.

	IFA		IFB		RSV		HPIV		HCoV		HMPV		HRV		COVID-19	
	OR (95%CI)	*P*	OR (95%CI)	*P*	OR (95%CI)	*P*	OR (95%CI)	*P*	OR (95%CI)	*P*	OR (95%CI)	*P*	OR (95%CI)	*P*	OR (95%CI)	*P*
Sex		NS		NS		NS		NS		NS		NS		NS		NS
Department	0.521 (0.364-0.745)	0.000		NS		NS	3.434 (1.150-10.250)	0.027		NS		NS	5.291 (1.193-23.460)	0.028	0.417 (0.261-0.667)	0.000
Age groups																
0-14yr		NS		NS		NS		NS		NS		NS	6.917 (1.858-25.751)	0.004		NS
15-24yr		NS		NS		NS		NS		NS		NS		NS		NS
25-59yr	-a			NS		NS		NS		NS		NS		NS		NS
60-69yr		NS		NS		NS		NS		NS		NS		NS		NS
≥70yr	0.617 (0.412-0.923)	0.034	0.310 (0.108-0.889)	0.029		NS		NS		NS		NS		NS	3.240 (1.905-5.511)	0.000
Clinical Symptoms																
Fever	1.566 (1.109-2.211)	0.011		NS		NS		NS		NS		NS		NS		NS
Cough	4.192 (1.974-8.904)	0.000		NS		NS		NS		NS		NS		NS	2.404 (1.062-5.443)	0.035
Sore throat	1.801 (1.237-2.624)	0.002		NS		NS		NS		NS		NS		NS		NS
Nasal congestion		NS	3.561 (1.202-10.551)	0.022		NS		NS		NS		NS		NS		NS
Shortness of breath		NS		NS		NS		NS		NS		NS		NS		NS
diarrhea		NS		NS		NS		NS		NS		NS		NS		NS
crackles	0.116 (0.028-0.486)	0.003		NS		NS		NS		NS		NS		NS	4.163 (2.397-7.231)	0.000

For each virus, an independent multivariate regression analysis was performed (i.e., whatever the virological result, positive or negative).

-a, Reference group; NS, no significant.

No significant differences were found in fever duration or white blood cell counts between virus-positive and negative patients (*P* > 0.05). HMPV cases showed the longest fever duration (3.81 ± 7.50 days), while IFB cases had the shortest (1.90 ± 1.03 days). For peak temperature, IFA, HPIV, HCoV, and HRV groups differed significantly from negatives (*P* < 0.05), with HMPV being highest (37.63 ± 0.80°C) and HRV lowest (36.33 ± 0.79°C). Hospital stays were significantly longer for IFA and IFB cases (*P* < 0.05), with RSV patients hospitalized longest (8.76 ± 5.54 days) and IFB shortest (6.69 ± 3.86 days). HPIV patients showed significant neutrophil count differences (*P* < 0.05), with IFB highest (5.39 ± 3.42 × 10⁹/L) and HPIV lowest (4.29 ± 0.49 × 10⁹/L). (Table 3 and 4).

**Table 3:** Clinical Indicators and Clinical Symptoms Associated with Respiratory Viruses Detection Positive.

Clinical indicators	IFA	IFB	RSV	HPIV	HCoV	HMPV	HRV	COVID-19
Duration of fever (days)	2.12 ± 1.49	1.90 ± 1.03	2.76 ± 3.05	2.05 ± 1.76	1.92 ± 2.02	3.81 ± 7.50	2.26 ± 2.02	2.27 ± 2.52
Maximum body temperature(°C)	37.67 ± 0.60	37.42 ± 0.75	37.53 ± 0.80	37.03 ± 0.91	36.81 ± 0.72	37.63 ± 0.80	36.33 ± 0.79	37.37 ± 0.91
Hospitalization period (days)	7.16 ± 4.21	6.69 ± 3.86	8.76 ± 5.54	8.53 ± 5.74	7.53 ± 4.03	8.30 ± 4.13	8.74 ± 3.53	8.30 ± 5.04
WBC(×10^9^/L)	7.09 ± 4.38	7.85 ± 4.38	7.62 ± 4.00	8.34 ± 5.80	7.76 ± 3.47	7.05 ± 3.34	8.52 ± 3.61	7.59 ± 3.23
NEUT(×10^9^/L)	5.16 ± 3.89	6.27 ± 4.44	5.45 ± 3.88	6.46 ± 5.53	5.83 ± 3.47	5.15 ± 3.18	6.55 ± 3.35	5.81 ± 3.90
Clinical Symptoms^a^								
Fever	140 (71.43)	20 (68.97)	22 (62.86)	22 (55.00)	19 (50.00)	19 (70.37)	19 (61.29)	60 (53.10)
Cough	188 (95.92)	27 (93.10)	32 (91.43)	36 (90.00)	33 (86.84)	26 (96.30)	30 (96.77)	105 (92.92)
Sore throat	59 (30.10)	11 (37.93)	5 (14.29)	6 (15.00)	4 (10.53)	4 (14.81)	4 (12.90)	23 (20.35)
Nasal congestion	16 (8.16)	5 (17.24)	1 (2.86)	2 (5.00)	2 (5.26)	1 (3.70)	2 (6.45)	7 (6.19)
Shortness of breath	13 (6.63)	1 (3.45)	3 (8.57)	5 (12.50)	5 (13.16)	2 (7.41)	3 (9.68)	20 (17.70)
diarrhea	2 (1.02)	2 (6.90)	2 (5.71)	0 (0.00)	0 (0.00)	0 (0.00)	0 (0.00)	7 (6.19)
crackles	2 (1.02)	1 (3.45)	1 (2.86)	1 (2.50)	1 (2.63)	2 (7.41)	2 (6.45)	30 (26.55)

^a^: case (%).

**Table 4:** Clinical Indicators and Clinical Symptoms Associated with Respiratory Viruses Detection Negative.

Clinical indicators	IFA	IFB	RSV	HPIV	HCoV	HMPV	HRV	COVID-19
Duration of fever (days)	2.17 ± 2.32	2.16 ± 2.31	2.15 ± 2.27	2.16 ± 2.31	2.16 ± 2.30	2.13 ± 2.08	2.16 ± 2.30	2.15 ± 2.28
Maximum body temperature(°C)	37.51 ± 0.72*	37.53 ± 0.71	37.53 ± 0.71	37.53 ± 0.71*	37.53 ± 0.51*	37.53 ± 0.71	37.53 ± 0.71*	37.54 ± 0.70
Hospitalization period (days)	8.11 ± 5.50*	8.16 ± 5.52*	8.14 ± 5.49	8.12 ± 5.49	8.14 ± 5.53	8.13 ± 5.52	8.12 ± 5.53	8.12 ± 5.53
WBC(×10^9^/L)	7.51 ± 3.61	7.46 ± 3.62	7.46 ± 3.62	6.62 ± 2.07	7.47 ± 3.65	7.46 ± 3.63	7.44 ± 3.63	7.46 ± 3.65
NEUT(×10^9^/L)	5.32 ± 3.40	5.39 ± 3.42	4.39 ± 0.96	4.29 ± 0.49*	5.39 ± 3.45	5.39 ± 3.44	5.36 ± 3.43	5.38 ± 3.44
Clinical Symptoms^#^								
Fever	886 (58.37)	1006 (59.77)	1004 (59.83)	1004 (60.05)	1007 (60.12)	1007 (59.79)	1007 (59.90)	966 (60.44)
Cough	1315 (87.26)	1474 (88.05)	1471 (88.14)	1467 (88.21)	1470 (88.24)	1477 (88.13)	1473 (88.10)	1398 (87.87)
Sore throat	220 (14.34)^*^	268 (17.03)^*^	274 (17.47)	273 (17.43)	275 (17.57)	275 (17.46)	275 (17.48)	256 (17.09)
Nasal congestion	50 (3.56)^*^	61 (3.91)	65 (4.18)	64 (4.12)	64 (4.12)	65 (4.16)	64 (4.10)	59 (3.96)
Shortness of breath	139 (9.87)	151 (9.65)	149 (9.56)	147 (9.45)	147 (9.45)	150 (9.58)	149 (9.53)	132 (8.85)^*^
diarrhea	43 (3.05)	45 (2.88)	43 (2.76)	45 (2.89)	45 (2.89)	45 (2.87)	45 (2.88)	38 (2.55)
crackles	117 (8.32)^*^	118 (7.56)	118 (7.59)	118 (7.60)	118 (7.60)	117 (7.49)	117 (7.50)	89 (5.98)^*^

* : Significant differences between case patients positive and negative for the specific virus (P < 0.05).

# : case (%).

## Discussion

Viruses are the primary pathogens responsible for SARI, with high morbidity and mortality, particularly among children under five years old [8]. Understanding the etiology and epidemiology of respiratory viruses is crucial for the prevention and control of SARI [9,10]. This study monitors 11 common respiratory viruses to better characterize the epidemiological features of SARI in Shandong, China.

The overall infection rate of respiratory viruses in SARI patients was 28.96% (501/1,730) in this study. This finding falls within the range reported in previous studies (24.5–38.7% in other Chinese regions; 41.2–72.3% in Western countries) [11–13], These findings reflect SARI’s geographic variation and etiological diversity, with IFA and COVID-19 being the most prevalent pathogens – consistent with reports from comparable clinical settings [7], while diverging from other epidemiological studies reporting distinct dominant viruses [14]. Our findings identify IFA as the primary causative agent of SARI, highlighting influenza vaccination’s crucial preventive role, especially for high-risk groups (infants, elderly, and those with comorbidities) [15].

The infection rate of IFA was highest in the 15–24 age group, which is inconsistent with previous research reports that concluded the prevalence of influenza virus increases with age [16]. This may be related to the fact that the 15–24 age group frequently participates in school and social activities, which accelerates the transmission of IFA respiratory virus [17]. COVID-19 emerged as the second most prevalent respiratory virus, with peak infection rates in adults ≥70 years – likely due to age-related immune decline impairing viral clearance, increasing viral load and prolonging infection [18]. HPIV showed the third highest infection rate in this study, predominantly affecting 25–59 year-olds, establishing it as a significant SARI cause. This pattern reflects variations in viral transmission dynamics, including reproduction numbers, mutation rates, immune duration, and cross-immunity [19], these viruses exhibit diverse distribution patterns across different age groups, which may aid in determining appropriate patient care and disease diagnosis.

SARI shows marked seasonal trends, especially in temperate zones, with Northern Hemisphere respiratory virus activity peaking in winter (November-March) due to enhanced viral survival in cold/dry conditions and increased indoor crowding..This study also demonstrated a similar trend, with the number of detected respiratory pathogens remaining at a high level from 47^th^ week to 50^th^ week of 2023, peaking in 48^th^ week. IFA infections exhibit seasonality, with higher prevalence in winter and spring, consistent with findings from studies in Spain and the United States [20,21] as well as the Eastern Mediterranean region [22]. However, this pattern differs from that observed in southern China [23], indicating regional variations in viral circulation.

This study found COVID-19 predominated in early autumn 2023, while other respiratory pathogens showed initially low but rapidly increasing infection rates, suggesting post-pandemic immune decline may have increased susceptibility to alternating epidemics [24]. HPIV, HCoV, and HRV exhibited a significant epidemic trend in autumn, whereas RSV, HMPV, and IFB showed a pronounced prevalence in winter. In contrast, other viruses demonstrated relatively low infection rates and sporadic circulation patterns, which are consistent with the epidemiological characteristics observed in previous reports [25].

Viral co-infections are frequently observed in respiratory viruses [26,27]. In our study, IFA, RV, RSV, HPIV, and HCoV were the most frequently detected co-infecting pathogens. Notably, the co-infection rate in the 15–24 age group was significantly higher than in other age groups, which may be associated with the increased social activities and higher exposure risk in this population. Furthermore, existing studies suggest that co-infection with respiratory viruses may exacerbate disease severity [1].

In our study, the results showed that the most common clinical symptoms in patients with SARI were fever and cough, which is consistent with previous reports [28].Our data indicated that symptoms associated with IFA infection include fever, cough, sore throat, and lung rales. The proportion of patients with high fever at hospital admission was higher among influenza patients than in non-influenza patients [29]. This suggests that during the influenza season, high-fever patients should be first assessed for IFA infection and receive proactive treatment to reduce the likelihood of febrile seizures. Symptoms associated with IFB infection include nasal congestion, suggesting that IFB infection is more likely to cause nasal congestion.

COVID-19 infection was significantly associated with pulmonary rales, whereas no significant association was observed between other respiratory viruses.These findings suggest that the presence of pulmonary rales may serve as a potential clinical indicator for COVID-19 infection in differential diagnosis. However, the clinical manifestations of respiratory viral infections are often similar and nonspecific [30,31]. Laboratory confirmation of pathogens through direct or indirect fluorescence assays provides critical diagnostic evidence to guide clinical decision-making, particularly given the nonspecific nature of respiratory infection symptoms.

This study has several limitations: (1) Exclusion of bacterial/atypical pathogens (e.g., Mycoplasma pneumoniae) may underestimate respiratory infection burden; (2) Limited pediatric inpatient numbers restrict analysis of childhood infections and may introduce selection bias; (3) The observation period was insufficient for comprehensive trend analysis.

## Conclusions

This study of 1,730 SARI cases in Shandong identified influenza A virus (39.12%) and COVID-19 (22.55%) as the predominant pathogens, demonstrating distinct winter seasonality and age-specific patterns. Influenza infection rates were highest among 15–24-year-olds, while COVID-19 posed the greatest risk for adults ≥70 years. Clinically, influenza primarily manifested with sudden-onset high fever and cough, whereas COVID-19 showed significant correlation with pulmonary rales. We recommend implementing prevention strategies tailored to specific age groups, seasons, and genders, while paying close attention to the characteristic clinical differences among various respiratory viral infections, to enhance the effectiveness of respiratory infection control measures.

## Supporting information

S1 File Comprehensive virological dataset of severe acute respiratory infections in Chinese hospitalized patients. Contains: Weekly distribution of cases and pathogen detection (Table A). Viral co-infection patterns and demographic characteristics (Table B).
(XLSX)
